# Is There a Diagnostic Miracle on the Horizon? Emerging Biomarkers in MASLD

**DOI:** 10.3390/jcm14176148

**Published:** 2025-08-30

**Authors:** Klaudyna Iwaszko-Sochal, Beata Kasztelan-Szczerbińska, Halina Cichoż-Lach

**Affiliations:** Department of Gastroenterology and Hepatology with Endoscopy Unit, Medical University of Lublin, Jaczewskiego 8, 20-954 Lublin, Poland; klaudyna.iwaszko92@gmail.com (K.I.-S.); halina.lach@umlub.edu.pl (H.C.-L.)

**Keywords:** MASLD, biomarkers, AGEs, sRAGE, PRO-C3, ADAPT, eicosanoids, fetuin-A, omics, precision medicine

## Abstract

The incidence and prevalence of metabolic dysfunction-associated steatotic liver disease (MASLD) are increasing, and, currently, the disease affects approximately 30% of the global population. Therefore, there is a growing need for widely available, patient-friendly, and reliable diagnostic tools. Our review is focused on the presentation and discussion of emerging biomarkers for evaluation and non-invasive detection of liver fibrosis in patients with MASLD, including glycation markers (AGEs/sRAGE), lipid mediators (eicosanoids), fetuin-A, collagen turnover markers (PRO-C3, ADAPT), and omic-based technologies. As reported recently, some of these parameters revealed high diagnostic accuracy in clinical trials, so they may be incorporated as key diagnostic tools in the future MASLD approach. Employment of such biomarkers may enable correct and quick identification of MASLD and/or MASH patients, as well as better monitoring of their treatment response. The development of precision medicine, driven by multiomics and individualized profiling, promises a rearrangement from the traditional “one size fits all” to tailoring targeted care, as environmental factors may have an even more relevant impact on MASLD pathogenesis in comparison with genetic predisposition. Nevertheless, to enable their widespread clinical use, novel biomarkers require further rigorous validation and standardized implementation in healthcare settings.

## 1. Introduction

Metabolic dysfunction-associated steatotic liver disease (MASLD) is the most common subtype of steatotic liver disease (SLD). According to current epidemiological data, MASLD affects approximately 25–30% of adults worldwide, with the highest prevalence rates reported in Latin America (~30%), the United States (~30–40%), and Europe (~20–25%) [1,2]. However, epidemiological projections predict further steady growth of its prevalence [3], due to an increasing tendency towards obesity, type 2 diabetes, and other cardiometabolic risk factors in the global population [4]. Despite intensive research, there is still no widely accepted and reliable diagnostic algorithm that would help to establish the proper diagnosis, assess the risk of advanced fibrosis, or the disease progression. A critical analysis of the available data, a comparison of methods, and identification of those with the greatest diagnostic potential in MASLD are essential to create modern and efficient solutions for patient management. Our review focuses on emerging non-invasive biomarkers for MASLD, with emphasis on their pathophysiological mechanisms, diagnostic accuracy, and potential clinical utility. We also highlight current limitations and outline future directions to support translation of these biomarkers into clinical practice.

## 2. MASLD—Definition, Pathogenesis, Classification

### 2.1. Current Nomenclature and Definition of MASLD

The term MASLD was introduced in 2023. It replaced the previous name, NAFLD (non-alcoholic fatty liver disease). According to the new definition, MASLD diagnosis requires both evidence of liver steatosis (via imaging or histology) and at least one of five metabolic risk factors (Table 1) [5].

The modification of the disease nomenclature and diagnostic criteria was finally approved after years of discussion and reports of close correlations between metabolic disorders and steatotic liver disease. The new terminology was based on a Delphi consensus determined a priori as a supermajority (67%) vote and provided by independent multinational experts of the European Association for the Study of the Liver (EASL) in collaboration with those of the American Association for the Study of Liver Diseases (AASLD) and the Latin American Association for the Study of the Liver (ALEH) [5]. NAFLD reclassification was intended to exclude the stigmatizing expression “fatty” and include cardiometabolic criteria for the confirmation of metabolic dysfunction. The consensus expanded the MASLD cohort into patients of lean or normal body mass presenting with metabolic factors, and reduced diversity in the disease definition. Renaming of NAFLD will better identify patients at risk of hepatic and cardiovascular metabolic complications and facilitate global communication to ensure consistency across clinical and research settings.

The populations covered by the traditional NAFLD criteria and the new MASLD criteria overlap almost completely. The confirmation comes from a study conducted on a random subgroup of 1016 individuals in Hong Kong, who were examined using magnetic resonance spectroscopy. It revealed no significant difference in the prevalence of NAFLD compared to MASLD (25.7% vs. 26.7%) [6]. The current diagnosis of MASLD requires the exclusion of alcohol abuse, defined as consumption above 140–350 g/week (20–30 g/day) in women and 210–420 g/week (30–60 g/day) in men [5]. The new consensus also acknowledges the possible conversion in the predominant disease course over time. Of note, some disease subtypes remained unchanged as alcohol-related liver disease (ALD), steatotic liver disease (SLD) of defined etiology (genetic background as Wilson disease, drug-induced, HCV-related, etc.), and cryptogenic form.

In our literature review, we use the new MASLD and MASH (metabolic dysfunction-associated steatohepatitis) terminology as recommended, but when citing original reports published before 2023, we follow the terms applied by their authors.

### 2.2. MASLD Relationship with Metabolic Syndrome and Alcohol Consumption

MASLD is a hepatic manifestation of systemic metabolic disorders, including disorders of lipid and glucose metabolism and chronic low-grade inflammation. This condition most often accompanies other disorders such as obesity, insulin resistance, type 2 diabetes, and dyslipidemia, and is the key manifestation of metabolic syndrome. Many studies have shown a higher prevalence of MASLD in patients with metabolic syndrome [7,8,9]. On the other hand, the results of a recent large meta-analysis showed that individuals with MASLD have a significantly higher risk of metabolic and systemic complications than those without this disease. An increased risk of developing type 2 diabetes (HR = 2.56; 95% CI: 2.10–3.13, *p* < 0.01), metabolic syndrome (hazard ratio—HR = 2.57; 95% confidence interval—CI: 1.13–5.85; *p* = 0.02), prediabetes (HR = 1.69; 95% CI: 1.22–2.35, *p* < 0.01), hypertension (HR = 1.75; 95% CI: 1.46–2.08, *p* < 0.01), cardiovascular disease (HR = 1.43; 95% CI: 1.27–1.60, *p* < 0.01), chronic kidney disease (HR = 1.38; 95% CI: 1.27–1.50, *p* < 0.01) and various types of cancer (HR = 1.54; 95% CI: 1.35–1.76; *p* < 0.01) was observed [10].

Obesity has long been recognized as a risk factor for the development of metabolic liver disease. Moreover, type 2 diabetes has been identified as the most important independent risk factor for the development of fibrosis and hepatocellular carcinoma (HCC) [11]. In the general population, individuals called “non-obese with MASLD” and “lean with MASLD” are estimated at 12.1% and 5.1%, respectively. Although MASLD is often linked to obesity, up to 40% of patients are non-obese [12]. To emphasize the role of coexisting risk factors, a new entity, MetALD (metabolic alcoholic liver disease), was proposed to reflect overlapping metabolic and alcohol-related liver injury [5]. It includes two subgroups: MetALD with a predominance of MASLD, when alcohol consumption is close to the lower limit (~20 g/day for women, ~30 g/day for men—140 g and 210 g per week, respectively), and MetALD with a predominance of alcohol-related liver disease (ALD), when high alcohol consumption is confirmed (50–60 g/day–350 g/week for women and 420 g/week for men) [13]. Both metabolic disorders and alcohol consumption have been shown to lead to common mechanisms of liver damage, such as oxidative stress, mitochondrial and endoplasmic reticulum damage, abnormal lipid and bile acid metabolism [14]. In MetALD, these mechanisms overlap and reinforce each other, causing faster progression of fibrosis and liver function impairment. In alcohol users, coexisting metabolic syndrome increases the risk of fibrosis up to 3.7-fold [15], which confirms a synergistic negative effect between increased body mass index (BMI) and alcohol misuse [16,17,18]. In a multicenter study involving two large groups of subjects, the relative excess risk resulting from the interaction between BMI and alcohol intake was estimated at 5.58 (CI 1.09–10.1), and the synergy index at 2.89 (CI 1.29–6.47) [16].

### 2.3. Pathogenesis and Clinical Course of MASLD

The development of MASLD involves multiple molecular pathways, including lipid accumulation, oxidative stress, and inflammation. The turning point is the intracellular accumulation of triglycerides in lipid droplets, which can comprise 5–10% of liver mass [19]. Lipotoxicity triggers inflammation and causes mitochondrial dysfunction, which in turn activates programmed cell death and promotes ballooning degeneration of hepatocytes [20]. As a result, excessive extracellular matrix production and fibrosis occur [21]. Another contributing factor is gut dysbiosis, which increases intestinal permeability of lipopolysaccharides (LPS) and other toxic metabolites. These molecules enter the portal circulation and trigger inflammatory activation of Kupffer cells, leading to cytokine storm with subsequent hepatic stellate cells transdifferentiation as well as peripheral inflammatory cells recruitment to the liver. Such disturbances are often diet-related and may lead to disease progression even in individuals with normal body weight [22].

MASLD comprises two conditions: MASL (metabolic-associated liver steatosis) and MASH (metabolic-associated steatohepatitis), which are histologically distinct. MASL presents with macrovesicular steatosis accompanied by mild inflammation and no signs of hepatocyte destruction, while distinctive features of MASH include inflammation with ballooning degeneration of hepatocytes and fibrosis of varying severity [23,24]. It is not easy to estimate the real proportion of MASH cases among all individuals with MASLD. The majority of patients with MASLD may not show any clinical or laboratory features of the disease. The natural MASLD course proceeds to hepatic fibrosis and cirrhosis, but most patients die from cardiovascular complications or cancer of a different origin. As reported, only about 10% of patients with MASLD die from complications of liver disease [1]. In a prospective study of 5123 people with NAFLD, the 15-year risk of cirrhosis development was estimated at 3% [25]. Of note, in patients with MASH, the prevalence of advanced fibrosis rises substantially to 9–25% within 10 to 20 years of disease onset [26]. This underscores the urgent need for reliable tools in order to identify individuals at high risk of disease progression.

## 3. Diagnosis and Treatment of MASLD

### 3.1. Algorithms in MASLD

The currently recommended algorithm for the diagnosis and monitoring of MASLD includes non-invasive tests and laboratory parameters. Screening for MASLD in primary care is recommended for individuals with risk factors (excessive body weight, diabetes, etc.) and with elevated liver enzymes or signs of fatty liver on imaging. The FIB-4 (Fibrosis-4 index) test is the first-choice diagnostic tool. People with a FIB-4 score < 1.3 should be monitored every 1–3 years, a score of 1.3–2.6 requires imaging or treatment adjustment, while a score > 2.6 indicates a need for referral to a specialist center for further clinical reassessment, imaging evaluations, and sometimes even a liver biopsy is required [23,27].

### 3.2. Laboratory Tests (FIB-4, NFS, APRI) for MASLD Patients

The FIB-4, the most widely used non-invasive biochemical marker of liver fibrosis, is calculated based on easily available parameters such as age, ALT, AST, and platelet count [28]. Other commonly used scores include the AST to platelet ratio index (APRI) and NAFLD fibrosis score (NFS). More advanced diagnostic models are under development and will be discussed in the biomarker section [29].

### 3.3. Imaging Techniques for MASLD Patients

Validated imaging techniques include dynamic elastography—most commonly vibration-controlled transient elastography (VCTE, FibroScan^®^)—as well as other elastography-related procedures [30]. The primary parameter assessed is liver stiffness, expressed in kilopascals (kPa). Magnetic resonance (MR) elastography offers high diagnostic accuracy, especially in obese patients, but its main drawback is the cost. While standard ultrasound and computed tomography (CT) scanning can confirm hepatic steatosis, they are not reliable for fibrosis assessment. However, when combined with clinical parameters, they may support MASLD diagnosis without enabling full disease staging.

### 3.4. Treatment of MASLD Patients

For many years, there was no effective pharmacological treatment for the disease. Currently, lifestyle changes and measures to improve metabolic status remain the relevant basis for MASLD treatment. Intensive research is underway to develop effective agents for the treatment of all stages of the disease, including fibrosis.

Currently, resmetirom—a thyroid hormone receptor β (THR-β) agonist—is the only U.S. Food and Drug Administration (FDA)-approved medication recommended in MASLD with proven efficacy in reducing liver fibrosis. The phase III MAESTRO-NASH trial demonstrated its superiority over placebo at doses of 80 and 100 mg [31,32]. Many medications potentially beneficial for MASLD patients still do not have recommendations for the treatment of the disease. They include glucagon-like peptide 1 (GLP-1) agonists [27], statins [33], and sodium-glucose cotransporter inhibitors (SGLT2 inhibitors) [34,35]. Several other drugs are currently under investigation, such as aramchol—a stearyl-CoA (coenzyme A) desaturase modulator [36], cenicriviroc—the C-C chemokine receptor type 5 (CCR5) inhibitor [37], the peroxisome proliferator-activated receptor (PPAR) agonists–lanifibranor [38] and saroglitazar [39], acetyl-coenzyme A (CoA) carboxylase inhibitor–firsocostat [40], apoptosis signal regulator type 1 kinase (ASK1) inhibitor–selonsertib [41].

## 4. Biomarkers with Diagnostic Power in MASLD

### 4.1. AGEs and sRAGE

AGEs (advanced glycation end products) are the end products of glycation, and sRAGE (soluble receptors for AGE) are soluble receptors for these products circulating in the blood. The term esRAGE (endogenous secretory RAGE) is sometimes used interchangeably with sRAGE. It represents a splice variant that contributes to the overall sRAGE pool [42]. Some studies assess only esRAGE concentrations, which show a strong correlation with total sRAGE levels [43,44].

AGEs comprise chemically diverse compounds, including proteins, lipids, and nucleic acids. They are primarily formed via non-enzymatic glycation under hyperglycemic and oxidative conditions [45]. The reaction between carbonyl groups of reducing sugars and amino groups of macromolecules results in stable end-products [46]. The most commonly studied compounds include pentosidine, N-carboxymethyl lysine (CML), and N-carboxyethyl lysine (CEL) [45]. Research into their impact on MASLD is ongoing. Population studies have shown that higher concentrations of free CEL and lower levels of PB-CML (protein-bound N-carboxymethyl-lysine) and pentosidine (β = −0.115 and −0.059, respectively) are significantly associated with increased liver fat content [47]. In one study, AGE fluorescence successfully distinguished between mild and moderate liver steatosis with an AUC (area under the curve) of 0.76 [48]. Interestingly, the potential impact of exogenous AGEs present in processed foods on mortality has been investigated, but the results were inconclusive, so this topic remains open for further investigation [49,50]. The mechanism of the toxic impact of glycation products is multifaceted. The most important one incorporates binding to proteins such as collagen and elastin with alteration of their structure and function impairment, leading to reduced tissue elasticity, vessel wall stiffness, and tissue regeneration disorders [51,52]. Other pathogenic mechanisms include the influence on nitric oxide production, activation of oxidative stress with the initiation of pro-inflammatory cytokine production: TNF-α (tumor necrosis factor alpha), IL-6 (interleukine 6), IL-1β (interleukine 6), and activation of RAGE receptors. The latter ones are found in endothelial cells, smooth muscles, hepatocytes, and immune cells. Collectively, these mechanisms promote chronic inflammation—a major contributor to metabolic syndrome and its related conditions, including diabetes, atherosclerosis, and MASLD [46]. In addition, the AGE–RAGE interaction triggers MAPK and JAK/STAT signaling, which further amplifies oxidative stress and profibrotic pathways. They involve the activation of hepatic stellate cells with subsequent extracellular matrix deposition, resulting in progression to liver fibrosis [46,52,53]. Therefore, the balance between AGE accumulation and sRAGE availability represents a critical driver of metabolic inflammation and fibrogenesis in MASLD.

Glycated hemoglobin (HbA1c) was the first widely used AGE and remains a key marker in diabetes diagnosis and management [54]. Numerous studies have revealed links between AGEs and diabetes complications such as retinopathy and neuropathy [55,56,57]. Currently, attention is focused on the relationship between AGE and sRAGE and cardiovascular disease. A large meta-analysis linked elevated AGE levels with increased both all-cause mortality (HR = 1.05, 95% CI 1.01–1.09; *p* = 0.018) and cardiovascular deaths (HR = 1.08, 95% CI 1.01–1.14; *p* = 0.015) [57]. As reported, AGEs can also be involved in the pathogenesis of neurodegenerative disorders, cancer, infertility, and even COVID-19 [46].

Research on the role of AGEs and sRAGE in liver disease has been ongoing for several years [58,59]. It is widely accepted that high AGE levels correlate with greater oxidative stress, markers of inflammation, and the risk of fibrosis [57], while higher sRAGE levels lead to a reduction in inflammation by blocking the interaction between AGEs and RAGEs [58]. There is already strong evidence for the involvement of the AGE/sRAGE axis in the pathogenesis of steatotic liver disease. However, circulating AGE and sRAGE levels may be influenced by several confounding factors that limit their specificity for MASLD. Dietary patterns, particularly high intake of processed foods, can substantially increase AGE burden [49,50]. Age-related accumulation of AGEs has also been documented [45,57], while comorbidities such as diabetes, chronic kidney disease, and cardiovascular disease may alter both AGE and sRAGE concentrations [55,56,57]. Lifestyle factors, including smoking and low physical activity, were also shown to reduce sRAGE levels [60]. These variables should therefore be considered when interpreting biomarker data in clinical settings.

In one study [59], the ratio of glycation products to sRAGE was 4-fold higher in NAFLD cases versus the control group, and an elevated AGE/sRAGE ratio (>7.8 mmol/pmol) was associated with a 12-fold increase in NAFLD risk. The AGE/sRAGE ratio showed good ability to distinguish NAFLD from normal liver, as shown by ROC curves (AUC = 0.85) [59]. The above parameter may therefore create an interesting predictor for the future screening of metabolic liver disease. Another Chinese study involved 246 patients with MASLD divided into two groups as follows: obese (BMI ≥ 25 kg/m^2^) and non-obese (BMI < 25 kg/m^2^), and compared with 95 healthy individuals from a control group. The mean serum concentrations of AGE and esRAGE were significantly higher in patients with MASLD compared to the control group (median AGE in patients with MASLD: 13.53, CI: 7.96–18.69, vs. 9.84, CI: 5.94–12.91, *p* < 0.001 in the control group; median esRAGE for MASLD: 2.21, CI: 1.71–2.74 vs. 1.9, CI: 1.49–2.23, *p* < 0.001 in the control group). Among MASLD patients, obese individuals had higher esRAGE concentrations than non-obese individuals (median 2.28, CI 1.84–2.94 compared to 2.09, CI 1.64–2.58, respectively, *p* = 0.023), whereas AGE levels did not differ significantly between the subgroups (*p* = 0.363) [61]. Another study found that lower sRAGE levels were significantly associated with elevated ALT activity (OR = 1.69, CI 1.11–2.57, *p* = 0.014). It also revealed correlations between lifestyle factors—such as smoking duration, sedentary behavior, and intake of red and processed meat—and low sRAGE concentrations [62]. The population study [63] of 1088 adults showed that lower sRAGE levels were linked to a higher FIB-4 score. Participants with the lowest sRAGE concentrations (lower quartile) had a significantly lower risk of elevated FIB-4 scores compared to those in the highest quartile (OR = 0.56; 95% CI 0.37–0.84, *p* = 0.001), suggesting a potential association between sRAGE and the severity of liver fibrosis [63]. Moreover, specific genetic polymorphisms of RAGE-related genes have been proposed as predictors of NASH development [60].

AGE and sRAGE are promising biomarkers for the diagnosis and monitoring of MASLD. However, their wider use is limited by the relatively high cost related to the reference detection methods, such as liquid chromatography–tandem mass spectrometry (HPLC-MS/MS) [63]. Nevertheless, the clinical advantages of HbA1c demonstrate that AGE-based markers can become standard diagnostic tools. Selected studies of the significance of AGEs and sRAGE in MASLD are presented in Table 2.

### 4.2. Lipid Metabolites (Eicosanoids)

The arachidonic acid pathway produces a variety of bioactive lipids that participate in both the initiation and resolution of inflammation, the regulation of immune responses, and the maintenance of membrane and vascular integrity. Most of these compounds have been well known for decades, and their role in many diseases has been thoroughly investigated. “Lipidomics” is a relatively new term used in the context of liver research. It refers to extensive examinations of various lipid molecules such as fatty acids, sphingolipids, glycerophospholipids, glycerolipids, sterol lipids, etc., their related interactions, and metabolic pathways within biological systems [64]. Since alterations in lipid profiles are implicated in the pathogenesis of various metabolic disorders, lipidomics is used for the identification of molecular marks linked to the aforementioned conditions. Lipids are involved not only in cell-to-cell signaling, energy homeostasis, and metabolic regulation, but also in inflammatory response. Arachidonic acid is initially released from cell membranes by phospholipase A2 and then metabolized by cyclooxygenases (COX), lipoxygenases (LOX), and cytochrome P450 enzymes (CYP450). These reactions lead to the formation of prostaglandins and thromboxanes (products of COX activity), leukotrienes and hydroxylated arachidonic acid derivatives (HETE—effect of LOX activity), as well as epoxy eicosatrienoic acids (EET) formed in the CYP 450 pathway. In addition, under conditions of oxidative stress, non-enzymatic reactions occur and lead to the formation of 11-HETE (11-hydroxy-eicosatetraenoic acid) or 13-HODE (13-hydroxyoctadecadienoic acid). The eicosanoids formed in this way perform various, often opposing biological functions. Some of them, such as 5-HETE (5-hydroxy-eicosatetraenoic acid), 8-HETE (8-hydroxy-eicosatetraenoic acid), and leukotrienes, have pro-inflammatory effects and may exacerbate hepatocyte damage. Others, such as lipoxins and resolvins, have anti-inflammatory properties and promote the suppression of inflammation and reduce fibrosis. In NASH (non-alcoholic steatohepatitis), there is a predominance of pro-inflammatory LOX and non-enzymatic products, along with reduced availability of precursors, which indicates metabolic dysregulation and susceptibility to inflammatory reactions and fibrosis [65].

In liver disease, eicosanoids are produced by adipocytes, immune cells, and hepatocytes in response to stress, much the same as in other tissues. Various eicosanoid metabolites are being investigated for further use. Lipidomic studies in patients with MASLD have shown significant changes in the concentration of many oxylipins (i.e., fatty acid oxidation products), including eicosanoids [66]. As mentioned previously, 5-HETE, formed from arachidonic acid in the 5-lipoxygenase pathway, has strong pro-inflammatory effects, as well as 9-HODE (9-hydroxyoctadecadienoic acid), a product of linoleic acid oxidation, which is an indicator of oxidative stress. Eicosapentaenoic acid (EPA) and its derivative 7,17-DHDPA (7,17-dihydroxydocosapentaenoic acid) have anti-inflammatory effects and may reflect an attempt to the induction of repair mechanisms in the liver. Adrenic acid and 14,15-DIHETE (14,15-dihydroxyecotetraenoic acid) are associated with the activation of CYP and LOX pathways, and their levels correlate with the severity of fibrosis [65].

The predictive value of circulating lipid mediators in patients with MASLD arises from their direct involvement in inflammatory and fibrogenic pathways. COX-derived prostaglandins and thromboxanes promote hepatic inflammation and platelet activation. LOX-derived leukotrienes and HETEs amplify oxidative stress and neutrophil recruitment, while P450 epoxygenase products such as EETs exert protective vasodilatory and anti-inflammatory effects. An imbalance between pro-inflammatory eicosanoids and pro-resolving mediators (lipoxins, resolvins) has been consistently associated with disease progression [65,66,67,68,69]. These mechanistic insights underline the potential of lipidomic markers not only as diagnostic tools but also as targets for therapeutic modulation through drugs interfering with COX/LOX pathways or with pro-resolving lipid mediators [65,66,67,68,69].

The described compounds, along with other components of the eicosanoid profile, exhibit diagnostic and prognostic value, allowing for the evaluation of liver injury severity and treatment outcome prediction.

Some eicosanoids, including 11,12-DIHETE (11,12-dihydroxyeicosatetraenoic acid), tetranor 12-HETE (a metabolite of tetranor 12-hydroxy-eicosatetraenoic acid), a shortened metabolite with four fewer carbon atoms (formed as a result of β-oxidation), adrenic acid, and 14,15-DIHETE, show a significant association with the severity of liver fibrosis. Changes in the concentration of selected molecules allowed monitoring of the stage of fibrosis and reduction in collagen content with moderate accuracy [67].

From a diagnostic point of view, diagnostic panels based on eicosanoids, in which many of them are measured simultaneously, are the most valuable method. The obtained data are processed by a computer. A profile based on seven eicosanoids (including 5-HETE, EPA, and 9-HODE) has proven useful in predicting fibrosis improvement within 24 weeks of observation [67]. The study evaluated the effectiveness of selected eicosanoids as markers of liver fibrosis improvement (defined as a reduction of at least one grade on the NASH clinical research network [NASH CRN] scale). Arachidonic acid (AA) showed the highest predictive value among the individual markers, achieving an AUC = 0.67 (95% CI 0.51–0.70; *p* = 0.0359). Adrenic acid also performed well (AUC = 0.58; *p* = 0.0373), and 5-HETE and 7,17-DHDPA were less effective (AUC 0.54 and 0.56, respectively) and had *p*-values at the borderline of statistical significance. The aforementioned panel of seven eicosanoids showed significantly better predictive value than individual markers, achieving an AUC of 0.74 (95% CI 0.62–0.87) [67]. Another study [66] evaluated the concentrations of specific oxidized fatty acids (oxFA) in the plasma of NAFLD patients and their association with histopathological changes. Among the markers analyzed, only linoleic acid oxidation products—9-HODE (9-hydroxyoctadecadienoic acid), 13-HODE (13-hydroxyoctadecadienoic acid), 9-oxoODE (9-oxooctadecadienoic acid) and 13-oxoODE (13-oxooctadecadienoic acid)—showed significantly higher values in patients with NASH compared to patients with simple steatosis and normal biopsy results (*p* = 0.02; 0.01; 0.002 and 0.01, respectively). For example, the ratio of 9-HODE to its precursor was 0.72 (CI 0.46–1.08) in the NASH group compared to 0.44 (CI 0.28–0.89) in the simple fatty liver group. Those with NASH also had significantly higher levels of 9-oxoODE (0.24 compared with 0.13 mmol/mol plasma) and 13-oxoODE (0.16 compared with 0.11 mmol/mol). The oxFA to precursor ratios for the other compounds did not differ significantly between patients with simple steatosis and patients without histological changes [66].

These results indicate that oxidative stress plays an important role in the progression of MASLD to the inflammatory phenotype, but not in the initial disease phase of simple steatosis. Oxidized linoleic acid products may be potential non-invasive biomarkers of this transformation [66].

An innovative initiative, the ‘MASLD LIPIDOMICS SCORE’ project [69], developed specialized eicosanoid profiles composed primarily of hydroxylated fatty acids such as 5-, 8-, 11-, and 15-HETE. Despite methodological challenges, this approach seems promising. To ensure diagnostic relevance, compounds with low plasma concentrations or non-significant differences were excluded from the final analysis. As many as 77 compounds had to be examined. The final model was presented as a complex algorithm incorporating multiple variables. After appropriate refinement, it could be introduced as a non-invasive screening test [69]. As a conclusion, lipid profiles have potential value as indicators of transformation occurring in the liver in MASLD development, such as tissue remodeling, fibrosis, and oxidative stress.

Compared to individual markers, lipidomic profiles offer superior sensitivity and predictive performance. However, they have notable limitations, including susceptibility to preanalytical variability, high analytical costs, and the need for rigorous compound selection. Moreover, advanced bioinformatics tools are required to interpret the data, as compound identification is only the initial step in generating a meaningful diagnostic model [70]. Selected studies of the significance of eicosanoids and lipidomics in MASLD are presented in Table 3.

### 4.3. Fetuin-A

Fetuin-A is closely involved in the key aspects of lipid and glucose metabolism, making it a likely contributor to the development of MASLD, a condition related to metabolic dysregulation.

Fetuin-A is a hepatokine that contributes to MASLD pathogenesis primarily by promoting insulin resistance and amplifying TLR-4 (toll-like receptor 4)—mediated inflammatory signaling, thereby linking metabolic disturbances with hepatic steatosis and fibrosis [71,72,73,74]. The interaction of fetuin-A with TLR-4 on immune cells (macrophages) intensifies the inflammatory response by activating the NF-κB pathway and leads to the production of pro-inflammatory cytokines (TNF-α, IL-6) [75,76]. In addition, the above binding induces TLR4-dependent signaling in platelets, leading to their hyper-reactivity, aggregation, and an increased risk of thrombosis in patients with MASLD [77] as discussed further in this paper.

Many reports have confirmed fetuin-A association with MASLD. One of them reveald that fetuin-A concentration was significantly correlated with CAP (controlled attenuation parameter, a non-invasive indicator of liver steatosis measured during elastography) (r = 0.34; *p* = 0.02) and MASLD score (r = 0.49; *p* < 0.001), Its value above 702.89 ng/mL showed high sensitivity (82%) and specificity (90%) in the diagnosis of MASLD (AUC = 0.95) [71].

Another study reported higher levels of fetuin-A in patients with NAFLD compared to healthy individuals (324 ± 98 vs. 225 ± 75 mg/L, *p* < 0.001). The same study showed a significant decrease in the level of this protein after metformin therapy compared to the placebo group (−40 ± 47 vs. 15 ± 82 mg/L, *p* = 0.008) [72]. Significantly higher levels of fetuin-A in patients with NAFLD were confirmed in a meta-analysis of 30 studies (Standardized Mean Difference—SMD = 0.83, 95% CI 0.59 to 1.07, Z-score = 6.82, *p* < 0.001) [73]. A similar meta-analysis of 17 studies was conducted a year later, confirming the conclusions of the previous report (SMD = 0.43, 95% CI 0.22–0.63, *p* < 0.001) [75].

However, the relationship between fetuin-A levels and the progression of liver fibrosis remains unclear. Some studies suggest that fetuin-A may have a protective effect against fibrosis, while others associate it with profibrotic processes. A negative correlation between fetuin-A levels and fibrosis severity has been reported, which may suggest a potential protective role of this protein [76,78,79]. Fetuin-A concentration has been shown to correlate positively with platelet count (r = 0.19; *p* < 0.01) and negatively with NAFLD fibrosis score (r = –0.25; *p* < 0.01) and carotid intima-media thickness (r = –0.22; *p* < 0.01) [76]. These findings suggest that reduced fetuin-A levels may reflect progressive liver fibrosis.

In a study by Zhang et al. [75], a positive correlation was found between plasma fetuin-A levels and platelet aggregation in NAFLD patients. Plasma fetuin-A levels were closely correlated with platelet aggregation induced by ADP (adenosine diphosphate—a potent platelet-activating factor) in two different variants: 1 µM ADP (r = 0.48, *p* < 0.01) and 2 µM ADP (r = 0.39, *p* < 0.01). Furthermore, the platelet activation was effectively diminished by a specific antibody that neutralizes fetuin-A. The fact that fetuin-A can promote platelet activation leads to the conclusion about its potential ability to increase the thromboembolic risk among patients with MASLD.

In the future, fetuin-A may be considered as a marker of liver steatosis, but its role as a prognostic factor for disease progression and complications remains uncertain. Notably, this molecule can display a dual role: its levels rise in MASLD and may exacerbate metabolic disturbances, yet some studies suggest a protective effect against fibrosis [76,78,79].

Selected studies of the significance of fetuin-A in MASLD are presented in Table 4.

### 4.4. Collagen Turnover Markers (PRO-C3, ADAPT)

The first reports on the use of collagen biomarkers in the assessment of liver fibrosis appeared at the turn of the 20th and 21st centuries [78,80,81,82], but groundbreaking research on PRO-C3 (collagen neoepitope biomarker PRO-C3) and the ADAPT system (a PRO-C3-based score) has been published in the last 10–15 years [83,84,85].

PRO-C3 is a blood-based biomarker indicating active synthesis of type III collagen—a structural protein deposited during liver fibrosis and synthesized by activated myofibroblasts [83]. It reflects the release of specific collagen fragments formed during cleavage of N-terminal propeptides by the enzyme ADAM-TS2 (A Disintegrin and Metalloproteinase with Thrombospondin motifs type 2). In contrast to the traditional PIIINP (type III procollagen N-terminal propeptide) marker, which indicates both synthesis and degradation of type III collagen, PRO-C3 specifically reflects its new production. It appears that PRO-C3 does not play a role in pathogenesis and is only a by-product of collagen synthesis; elevated PRO-C3 levels reflect remodeling of the extracellular matrix caused by activation of stellate cells and TGF-β signaling, which accompanies the progressive stages of the disease [84,86,87,88]. Research has confirmed its strong association with both the level and pace of fibrosis progression [86].

The most intensively studied molecules also include C4M (a fragment of type IV collagen broken down by matrix metalloproteinases), PRO-C18L (procollagen type XVIII long form N-terminal propeptide), PIIINP (type III procollagen N-terminal propeptide), and TIMP-1 (tissue inhibitor of metalloproteinases-1). These molecules have been found to reflect the dynamics of fibrosis, and most of the available results concern this process in the course of MASLD.

A meta-analysis of 14 studies conducted in 2023 [87] showed that the assessment of PRO-C3 concentration alone is highly effective in identifying patients with advanced liver fibrosis (F2 and F3). The area under the ROC (AUROC) value obtained was 0.80 (95% CI 0.76–0.83). PRO-C3 levels increased with the progression of fibrosis (r = 0.50; *p* < 0.0001) and were independently associated with advanced fibrosis (OR = 1.05; 95% CI 1.02–1.08; *p* = 0.003) [88]. A similar result was obtained in another study: plasma PRO-C3 concentrations increased significantly with the severity of all NASH activity features and the degree of fibrosis. The AUROC values for the detection of significant fibrosis (F2–F4), advanced fibrosis (F3–F4), and NASH were 0.71, 0.73, and 0.74, respectively (*p* < 0.001 for all). PRO-C3 was also significantly elevated in patients with NAS (NAFLD activity score) ≥4 and independent of fibrosis stage. It was also significantly higher in patients with NASH compared to patients with simple steatosis (NAFL) (*p* < 0.001) [89].

To improve diagnostic accuracy, the ADAPT model was developed by combining PRO-C3 with clinical parameters such as age, diabetes status, and platelet count. This model outperformed traditional indices like APRI, FIB-4, NFS, and PRO-C3 alone [88,89]. A study comparing the effectiveness of non-invasive markers in the diagnosis of advanced fibrosis (F2–F4) demonstrated the superiority of the ADAPT algorithm over other serum-based scales [89]. In the model group, the AUROC value for ADAPT reached 0.855 and was higher than for APRI (0.73; *p* = 0.02), FIB-4 (0.78; *p* = 0.06), and NAFLD Fibrosis Score (0.78; *p* = 0.06). Similar results were obtained in the validation group, where ADAPT achieved an AUROC of 0.87 compared to APRI (0.78; *p* = 0.0005), FIB-4 (0.85; *p* = 0.32), and NFS (0.79; *p* = 0.02) [88].

Combining the Agile-4 score—which incorporates results of FibroScan^®^ elastography, ALT, AST, and platelet count—with the ADAPT model further enhanced diagnostic performance. The combination of the ADAPT and Agile-4 models in the form of a sequential algorithm showed the highest effectiveness in stratifying the risk of severe fibrosis (≥F3), achieving an AUROC value of 0.88 and maintaining a high predictive value in patients with a negative result [90]. These indicators demonstrated great value in the stratification of risk of liver fibrosis among MASLD patients, especially in ruling out those at low risk. This can be attributed to the excellent negative predictive performance of most bone turnover markers, with ADAPT and Agile-4 demonstrating negative predictive values (NPVs) of 0.972 and 0.992, respectively [90]. An algorithm based on PRO-C3 and ADAPT has also been shown to be effective in identifying patients at risk of advanced liver fibrosis in the course of ALD (alcoholic liver disease): PRO-C3 achieved an AUROC of 0.85 (95% CI: 0.79–0.90), and the ADAPT algorithm further increased diagnostic accuracy (AUROC 0.88; 95% CI: 0.83–0.93) [91]. Selected studies of the significance of PRO-C3 and ADAPT in MASLD are presented in Table 5.

### 4.5. Omics Technologies

The concept of ‘omics’ or ‘multiomics’ refers to a modern diagnostic approach that integrates multiple biological data layers—genomics, transcriptomics, epigenomics, proteomics, and metabolomics. Data from multiple omics layers as genetic variants and polymorphisms (genomics), gene expression profiles based on mRNA and miRNA (transcriptomics), DNA methylation patterns (epigenomics), protein biomarkers (proteomics), and metabolic alterations like changes in lipid levels (metabolomics), are included. Omics approaches are the result of years of research on single biomarkers, whose individual application offers only limited insights. Combining them through multiomic analysis provides a powerful tool for high-precision patient phenotyping and holds promise for diagnostics, treatment selection, and prognosis [92].

The application of multiomic diagnostics became feasible due to the development of specialized databases. Genome sequencing has provided information on genetic variants [93], while the HUPO project (Human Proteome Organization) [94] analyzed proteins in the body and compared them with transcriptomic data to determine their sites of synthesis and function. The Human Metabolome Database (HMDB) [95], developed within the Human Metabolome Project, remains the leading reference for studies on small-molecule metabolites in the human body.

Omics-based diagnostics rely on integrating large-scale laboratory data with machine learning, artificial intelligence, and cloud computing to construct sophisticated mathematical models. The ultimate goal is to develop tools capable of analyzing a single patient sample—such as blood, for example—to simultaneously assess multiple parameters and generate a comprehensive phenotypic profile through an analysis process [92]. The process begins with data collection using advanced platforms such as Somascan or Olink, which can detect thousands of proteins or metabolites in parallel. Subsequent steps involve computer analysis based on multiple trials. Collected data is extracted for use in future tests, and a model containing specific parameters is constructed based on this data. Then, the model must be verified and compared with conventional tests such as APRI, FIB-4, NFS, or transaminase activity.

In the study by Luo et al. [96], researchers analyzed 1305 serum proteins and developed two diagnostic models: one incorporating four proteins and another using twelve. Both outperformed conventional markers such as FIB-4 and NFS in terms of sensitivity and specificity for fibrosis detection in SLD. In another study [97], models based on proteins, enzymes, and genetic data were constructed. The best results were obtained for combined methods compared to every single one.

Multiomic platforms also support weighted gene co-expression network analysis (WGCNA). This method helps to identify clusters of genes (modules) that share similar expression profiles and are linked to specific clinical traits and potentially have regulatory functions [98]. This approach makes it possible to link groups of genes to phenotypic traits, tissues, or disease states [99,100].

Adaptation of multi-omics data technologies has enormous potential and surely helps in both better explanation of the complex MASLD pathogenesis at different molecular and metabolic levels and the development of clinical precision medicine in the field. Incorporation of data from various omics technologies allows a profound disease outlook. Sophisticated examinations of molecules in biological systems are aimed not only to reveal novel biomarkers for MASLD staging and complication risk stratification, but also to discover solutions that will help to create targeted and effective patient management. An abundance of omics technologies has enabled the discovery of the distinctive nature of MASLD to never-before performed extent, providing a comprehensive approach to molecular regulatory pathways and involving several organs’ interactions in the course of the disease. Indeed, they deepened our knowledge of MASLD.

Omics-based approaches hold great promise for the future of diagnostics, but they still face significant challenges, including high costs, time-consuming validation processes, and the need to tailor models for diverse populations. Researchers must cope with drawing reasonable and logical insights from a rapidly increasing amount of data. The identification of reliable protein and genetic biomarkers requires decades of research by numerous independent teams worldwide. However, there is growing optimism that these efforts will eventually lead to the development of simple, non-invasive, and clinically reliable tools for diagnosing and predicting liver fibrosis and MASLD clinical course monitoring.

It should be emphasized that omics-derived biomarkers do not necessarily reflect causal mechanisms in MASLD pathogenesis. Many lipidomic or proteomic signatures represent correlations with disease severity or progression, but may act merely as surrogate indicators of metabolic or inflammatory changes rather than direct drivers of hepatic injury [66,69,93,97]. Therefore, while such markers improve diagnostic performance, their biological interpretation requires caution, and further validation is needed before clinical translation.

The progress in multi-omics diagnostics concurrent with the artificial intelligence revolution will facilitate the future expansion of advanced algorithms in MASLD and help to introduce them to clinical practice. Nevertheless, large-scale validation studies and technological optimization are required to complete this process.

## 5. Conclusions and Prospects in MASLD Evaluation

The increase in the incidence of MASLD requires the development of effective, non-invasive, and easily accessible diagnostic tools to identify patients at risk of developing and progressing to liver fibrosis. Currently available data indicate the usefulness of markers such as PRO-C3,

Other biomarkers, such as AGEs/sRAGE, lipidomic, and multi-metabolic profiles, show potential for future assessment of MASLD severity and predicting disease progression. Among these, the ADAPT algorithm and combined lipid panels appear particularly promising, achieving an AUROC value of 0.88 in the detection of advanced fibrosis. The ongoing development of omics technologies and the integration of multifactorial data using artificial intelligence offer hope for the creation of complex but easy-to-use tools that could revolutionize MASLD diagnostics in the future.

Emerging evidence supports the diagnostic and prognostic utility of multi-marker panels, which may help identify patients with the highest risk of fibrosis progression and guide decisions on their need for intensified management. Single biomarkers offer limited diagnostic value, but their combined analysis using modern bioinformatics tools may significantly improve the accuracy and clinical relevance of non-invasive MASLD assessment.

Despite promising results, new biomarkers have a number of limitations.

AGEs and sRAGE are attractive due to their association with oxidative stress and fibrosis, but the tests are highly heterogeneous (ELISA vs. fluorescence-based detection), and there are no widely accepted thresholds, which hinders their application [46,53,58,59].

Although fetuin-A has been proposed as a new non-invasive biomarker for MASLD [71,72,73,74,75,76,77,78,79], its clinical utility remains uncertain. Reports on associations are inconsistent, with some studies showing elevated levels in MASLD [71,73] and others an inverse correlation with fibrosis progression [76].

The use of fetuin-A in clinical practice is complicated by the strong influence of comorbid metabolic disorders, such as obesity and insulin resistance, which reduce its specificity in MASLD [71,72,73]. In addition, most of the available studies are clinical trials with a control group, which limits the generalizability of the results.

Although PRO-C3 and ADAPT show promising diagnostic accuracy [88,89,90,91,92], their clinical application is limited by the lack of standard cut-off values, the relatively high cost of ELISA-based tests compared to routine indicators such as FIB-4, and limited availability outside research centers.

Despite the excellent diagnostic value of metabolomic and lipidomic signatures [63,70,92,96], their implementation in clinical practice is limited by high costs, the need for specialized mass spectrometry platforms, and a lack of standardization between laboratories [70,92,101].

Considering all the biomarkers described, many studies include only small, selected groups of patients, and large cohorts in everyday practice are lacking. Furthermore, despite their high value, biomarkers are not always tested directly, which makes comparison difficult—a proper comparative assessment needs to be performed.

The most influential agencies, including the Food and Drug Administration (FDA) and European Medicines Agency (EMA), have not yet approved most of these biomarkers as diagnostic tools.

Future research should focus on integrating multiomic approaches—including lipidomics, proteomics, and genomics—to develop robust prognostic algorithms that reflect the complexity of MASLD pathogenesis [64,65,100,102]. In comparison to single analytes, the development of multimarker panels provides greater diagnostic and prognostic power, potentially improving both sensitivity and specificity across the disease spectrum [96,101]. Although multiomic approaches have identified complex biomarker panels with promising diagnostic accuracy, they often represent only associative patterns rather than actual pathways. This distinction highlights the need for large-scale validation studies and studies to confirm their pathophysiological relevance [65,93,101].

Based on currently available evidence and research capabilities, the implementation of precise diagnostic or therapeutic algorithms based solely on new biomarkers is not currently possible. Therefore, in our updates, we direct clinicians’ attention to the two-step pathway outlined in the guidelines [23,27,30] and summarize where biomarker panels such as PRO-C3/ADAPT could be placed after completion of validation and implementation studies [86,89,90,91].

Once new biomarkers become available for widespread use in clinical practice, efforts should focus on simplifying procedures that could be easily implemented in standard hospital laboratories or outpatient settings [84,88,90]. Another key step is the systematic assessment of cost-effectiveness, as many advanced biomarker tests remain expensive, technically demanding, and broadly unavailable. Comparative economic evaluations will be essential to balance diagnostic accuracy and affordability, particularly in resource-limited settings. Therefore, large-scale, prospective validation studies with long-term follow-up are needed, not only to confirm diagnostic and prognostic utility but also to determine whether biomarker-based strategies can reduce the costs associated with late diagnosis, disease progression, and advanced liver complications. Ultimately, combining clinical efficacy with economic feasibility will be crucial for the widespread use of biomarker-based approaches in MASLD.

The development of modern analytical methods has led to a shift away from conventional treatment strategies toward “precision medicine”—an approach tailored to individual variants of genetics, metabolism, environment, and clinical status of the patient. The goal of this personalized approach is to select the most appropriate therapy for a specific patient—the only one that is more effective and safer than treatment relying on averaged population data [100,102]. By 2030, precision medicine is expected to become a standard in clinical care through the routine application of multiomics, microbiome profiling, and comprehensive metabolic monitoring [103].

There is strong evidence that medicine is increasingly shifting toward personalization; however, large-scale validation studies and standardization efforts are still required to translate emerging biomarkers into routine clinical practice.

Therefore, answering in brief to the title question of the paper: advances in MASLD diagnostic approach are significant, but no single breakthrough qualifies as the diagnostic miracle so far, and, also, clinical validation takes time.

## Figures and Tables

**Table 1:** Comparison of diagnostic criteria for MASLD and NAFLD *. Based on [5].

MASLD	NAFLD
Recognize steatosis (imaging, biomarkers, biopsy)	Recognize steatosis (imaging, biomarkers, biopsy)
Exclude other causes of steatosis and alcohol abuse: ≤2 drinks per day for men (30 g of alcohol) ≤1 drink per day for women (20 g of alcohol)	Exclude other causes of steatosis and alcohol abuse: ≤2 drinks per day for men (30 g of alcohol) ≤1 drink per day for women (20 g of alcohol)
Confirm at least 1 of the following risk criteria: - BMI over 25 kg/m^2^ (23 in Asians) - Waist circumference over 94 ♂/80 ♀ cm - BP over 130/85 mmHg or known hypertension - Plasma triglycerides over 150 mg/dL (1.7 mmol/L) or specific treatment - Plasma HDL <40 mg/dL (1 mmol/L) for men or <50 mg/dL (1.3 mmol/L) for women or specific treatment - Prediabetes state (IFG > 100 mg/dL, IGT >140 mg/dL) or recognized type 2 diabetes	

* BMI—body mass index; BP—blood pressure; IFG—impaired fasting glucose, IGT—impaired glucose tolerance; MASLD—metabolic dysfunction-associated steatotic liver disease; NAFLD—non-alcoholic fatty liver disease.

**Table 2:** Selected studies of the significance of AGEs and sRAGE in MASLD *.

Parameter	Study Group	Key Findings	Reference
Free CEL, PB-CML, pentosidine	Cohort on Diabetes and Atherosclerosis Maastricht (CODAM) n = 505	Higher free CEL and lower PB-CML and pentosidine significantly associated with increased liver fat content (β = −0.115 and −0.059)	[47]
AGE fluorescence	NAFLD n = 909, ALD n = 169 healthy controls n = 766	Differentiated mild vs. moderate steatosis with AUC = 0.76	[48]
AGE/sRAGE ratio	NAFLD adults n = 58 healthy controls n = 58	Ratio 4-fold higher in NAFLD; value >7.8 mmol/pmol increased NAFLD risk 12-fold; AUC = 0.85	[59]
Serum AGE and esRAGE	NASH n = 103, non-NASH NAFLD n = 143, normal liver histology n = 93	Higher AGE and esRAGE in NAFLD vs. controls; higher esRAGE in obese NAFLD vs. non-obese (*p* = 0.023)	[60]
Low sRAGE	MASLD n = 246 healthy controls n = 95	Associated with elevated ALT (OR = 1.69; CI 1.11–2.57; *p* = 0.014); correlated with smoking, sedentary lifestyle, red meat intake	[61]
Low sRAGE quartile	NAFLD n = 266 Normal liver n = 457	Linked to higher FIB-4 score; OR = 0.56 (95% CI 0.37–0.84; *p* = 0.001)	[62]

* AGEs—advanced glycation end products, CEL—N-carboxyethyl lysine, esRAGE—endogenous secretory receptors for AGE, PB-CML—protein-bound N-carboxymethyl-lysine, sRAGE—soluble receptors for AGE.

**Table 3:** Selected studies of the significance of eicosanoids and lipidomics in MASLD *.

Parameter	Study Group	Key Findings	Reference
5-HETE, 9-HODE	MASLD	Pro-inflammatory effect; markers of oxidative stress	[66,67,68]
EPA, 7,17-DHDPA	MASLD	Anti-inflammatory; associated with repair mechanisms	[66]
Adrenic acid, 14,15-DIHETE	MASLD	Correlated with fibrosis severity	[66,68]
Panel of 7 eicosanoids (incl. 5-HETE, EPA, 9-HODE)	MASLD	Predicted fibrosis improvement after 24 weeks; AUC = 0.74 (95% CI 0.62–0.87)	[68]
Oxidized linoleic acid derivatives (9-HODE, 13-HODE, 9-oxoODE, 13-oxoODE)	NAFLD	Significantly higher in NASH vs. steatosis and controls (*p* = 0.002–0.02); e.g., ratio 9-HODE/precursor 0.72 vs. 0.44	[69]
MASLD LIPIDOMICS SCORE	MASLD	Algorithm based on hydroxylated fatty acids; promising but requires validation	[70]

* DHDPA—dihydroxydocosapentaenoic acid, DIHETE—dihydroxyecotetraenoic acid, EPA—eicosapentaenoic acid, HETE—hydroxy-eicosatetraenoic acid, HODE—13-hydroxyoctadecadienoic acid, oxoODE—oxooctadecadienoic acid.

**Table 4:** Selected studies of the significance of fetuin-A in MASLD.

Parameter	Study Group	Key Findings	Reference
Serum fetuin-A	biopsy-proven NAFLD n = 82	No significant correlation with fibrosis stage (F0–F4); trend toward lower levels in F1 vs. F0 (*p* = 0.067)	[76]
Fetuin-A levels	Various clinical studies	Ambiguous results: some show higher concentrations in MASLD [71,72,73], others inverse association with fibrosis progression [76]	[71,72,73,74,75,76]
Influence of comorbidities	MASLD patients with obesity/IR	Specificity reduced due to strong metabolic confounding	[71,72,73,74,75,76,77,78]

**Table 5:** Selected studies of the significance of PRO-C3 and ADAPT in MASLD *.

Parameter	Study Group	Key Findings	Reference
PRO-C3	MASLD with fibrosis	Serum levels correlate with fibrosis stage; independent predictor of advanced fibrosis	[86,88]
ADAPT score (Age, Diabetes, PRO-C3, Platelets)	Validation cohorts	AUC for advanced fibrosis 0.86–0.87, superior to FIB-4 and NFS	[89]
Sequential algorithm (PRO-C3 + ADAPT)	Clinical studies	Improved stratification of high-risk patients; better performance than individual markers	[90,91]
Comparison with conventional scores	MASLD/NAFLD cohorts	Outperformed APRI, FIB-4, NFS in sensitivity and specificity for fibrosis detection	[88,89,90]
Cut-off values	Various studies	No universally accepted cut-offs; thresholds vary depending on cohort and assay used	[86,87,88,89,90,91]

* ADAPT—an algorithm incorporating PRO-C3, APRI—the AST to platelet ratio index, FIB-4—Fibrosis-4 index, NFS—NAFLD fibrosis score, PRO-C3—collagen neoepitope biomarker PRO-C3.

## Data Availability

All data are included in the main text.

## Abbreviations

The following abbreviations are used in this manuscript:

AA	Arachidonic Acid
AASLD	American Association for the Study of Liver Diseases
ADAM	A Disintegrin and Metalloproteinase
ADAPT	Age, Diabetes, PRO-C3, Platelets (fibrosis prediction model)
AGE	Advanced Glycation End-products
ALD	Alcoholic Liver Disease
ALT	Alanine Aminotransferase
APRI	AST to Platelet Ratio Index
AST	Aspartate Aminotransferase
AUC	Area Under the Curve
AUROC	Area Under the Receiver Operating Characteristic Curve
CEL	N-Carboxyethyllysine
CML	N-Carboxymethyllysine
FIB-4	Fibrosis-4 Index
GGT	Gamma-glutamyl Transferase
IMT	Intima-Media Thickness
LDL	Low-Density Lipoprotein
MASH	Metabolic Dysfunction-Associated Steatohepatitis
MASLD	Metabolic Dysfunction-Associated Steatotic Liver Disease
NAFL	Non-Alcoholic Fatty Liver
NAFLD	Non-Alcoholic Fatty Liver Disease
NPV	Negative Predictive Value
PB-CML	Protein-Bound Carboxymethyllysine
PRO-C3	N-terminal pro-peptide of type III collagen
T2DM	Type 2 Diabetes Mellitus
sRAGE	Soluble Receptor for Advanced Glycation End-products

## References

[1] Younossi Z.M., Koenig A.B., Abdelatif D., Fazel Y., Henry L., Wymer M. (2016). Global epidemiology of nonalcoholic fatty liver disease-Meta-analytic assessment of prevalence, incidence, and outcomes. Hepatology.

[2] Eskridge W., Cryer D.R., Schattenberg J.M., Gastaldelli A., Malhi H., Allen A.M., Noureddin M., Sanyal A.J. (2023). Metabolic Dysfunction-Associated Steatotic Liver Disease and Metabolic Dysfunction-Associated Steatohepatitis: The Patient and Physician Perspective. J. Clin. Med..

[3] Le M.H., Yeo Y.H., Li X., Li J., Zou B., Wu Y., Ye Q., Huang D.Q., Zhao C., Zhang J. (2022). 2019 Global NAFLD Prevalence: A Systematic Review and Meta-analysis. Clin. Gastroenterol. Hepatol..

[4] Stefan N., Yki-Järvinen H., Neuschwander-Tetri B.A. (2025). Metabolic dysfunction-associated steatotic liver disease: Heterogeneous pathomechanisms and effectiveness of metabolism-based treatment. Lancet Diabetes Endocrinol..

[5] Rinella M.E., Lazarus J.V., Ratziu V., Francque S.M., Sanyal A.J., Kanwal F., Romero D., Abdelmalek M.F., Anstee Q.M., Arab J.P. (2023). A multisociety Delphi consensus statement on new fatty liver disease nomenclature. Hepatology.

[6] Song S.J., Lai J.C., Wong G.L., Wong V.W., Yip T.C. (2023). Can we use old NAFLD data under the new MASLD definition?. J. Hepatol..

[7] Chen S.H., He F., Zhou H.L., Wu H.R., Xia C., Li Y.M. (2011). Relationship between nonalcoholic fatty liver disease and metabolic syndrome. J. Dig. Dis..

[8] Hsiao P.-J., Kuo K.-K., Shin S.-J., Yang Y.-H., Lin W.-Y., Yang J.-F., Chiu C.-C., Chuang W.-L., Tsai T.-R., Yu M.-L. (2007). Significant correlations between severe fatty liver and risk factors for metabolic syndrome. J. Gastroenterol. Hepatol..

[9] Uchil D., Pipalia D., Chawla M., Patel R., Maniar S., Narayani, Juneja A. (2009). Non-alcoholic fatty liver disease (NAFLD)-the hepatic component of metabolic syndrome. J. Assoc. Physicians India.

[10] Chan K.E., Ong E.Y.H., Chung C.H., Ong C.E.Y., Koh B., Tan D.J.H., Lim W.H., Yong J.N., Xiao J., Wong Z.Y. (2024). Longitudinal Outcomes Associated with Metabolic Dysfunction-Associated Steatotic Liver Disease: A Meta-analysis of 129 Studies. Clin. Gastroenterol. Hepatol..

[11] Ajmera V., Cepin S., Tesfai K., Hofflich H., Cadman K., Lopez S., Madamba E., Bettencourt R., Richards L., Behling C. (2023). A prospective study on the prevalence of NAFLD, advanced fibrosis, cirrhosis and hepatocellular carcinoma in people with type 2 diabetes. J. Hepatol..

[12] Ye Q., Zou B., Yeo Y.H., Li J., Huang D.Q., Wu Y., Yang H., Liu C., Kam L.Y., Tan X.X.E. (2020). Global prevalence, incidence, and outcomes of non-obese or lean non-alcoholic fatty liver disease: A systematic review and meta-analysis. Lancet Gastroenterol. Hepatol..

[13] Dunn N., Al-Khouri N., Abdellatif I., Singal A.K. (2025). Metabolic Dysfunction and Alcohol-Associated Liver Disease: A Narrative Review. Clin. Transl. Gastroenterol..

[14] Gratacós-Ginès J., Ariño S., Sancho-Bru P., Bataller R., Pose E. (2024). MetALD: Clinical aspects, pathophysiology and treatment. JHEP Rep..

[15] Pose E., Pera G., Torán P., Gratacós-Ginès J., Avitabile E., Expósito C., Díaz A., Graupera I., Rubio A.B., Ginès P. (2021). Interaction between metabolic syndrome and alcohol consumption, risk factors of liver fibrosis: A population-based study. Liver Int..

[16] Hart C.L., Morrison D.S., Batty G.D., Mitchell R.J., Davey Smith G. (2010). Effect of body mass index and alcohol consumption on liver disease: Analysis of data from two prospective cohort studies. BMJ.

[17] Naveau S., Giraud V., Borotto E., Aubert A., Capron F., Chaput J.C. (1997). Excess weight risk factor for alcoholic liver disease. Hepatology.

[18] Boyle M., Masson S., Anstee Q.M. (2018). The bidirectional impacts of alcohol consumption and the metabolic syndrome: Cofactors for progressive fatty liver disease. J. Hepatol..

[19] Neuschwander-Tetri B.A., Caldwell S.H. (2003). Nonalcoholic steatohepatitis: Summary of an AASLD Single Topic Conference. Hepatology.

[20] Ten Hove M., Pater L., Storm G., Weiskirchen S., Weiskirchen R., Lammers T., Bansal R. (2020). The hepatic lipidome: From basic science to clinical translation. Adv. Drug Deliv. Rev..

[21] Li Y., Yang P., Ye J., Xu Q., Wu J., Wang Y. (2024). Updated mechanisms of MASLD pathogenesis. Lipids Health Dis..

[22] Jiménez-González C., Alonso-Peña M., Argos Vélez P., Crespo J., Iruzubieta P. (2025). Unraveling MASLD: The Role of Gut Microbiota, Dietary Modulation, and AI-Driven Lifestyle Interventions. Nutrients.

[23] Rinella M.E., Neuschwander-Tetri B.A., Siddiqui M.S., Abdelmalek M.F., Caldwell S., Barb D., Kleiner D.E., Loomba R. (2023). AASLD Practice Guidance on the clinical assessment and management of nonalcoholic fatty liver disease. Hepatology.

[24] Brown G.T., Kleiner D.E. (2016). Histopathology of nonalcoholic fatty liver disease and nonalcoholic steatohepatitis. Metabolism.

[25] Allen A.M., Therneau T.M., Ahmed O.T., Gidener T., Mara K.C., Larson J.J., Canning R.E., Benson J.T., Kamath P.S. (2022). Clinical course of non-alcoholic fatty liver disease and the implications for clinical trial design. J. Hepatol..

[26] Kumar R., Priyadarshi R.N., Anand U. (2020). Non-alcoholic fatty liver disease: Growing burden, adverse outcomes and associations. J. Clin. Transl. Hepatol..

[27] Tacke F., Horn P., Wong V.W.-S., Ratziu V., Bugianesi E., Francque S., Zelber-Sagi S., Valenti L., Roden M., Schick F. (2024). EASL-EASD-EASO Clinical Practice Guidelines on the management of metabolic dysfunction-associated steatotic liver disease (MASLD). J. Hepatol..

[28] Vallet-Pichard A., Mallet V., Nalpas B., Verkarre V., Naplas A., Dhalluin-Venier V., Fontaine H., Pol S. (2007). FIB-4: An inexpensive and accurate marker of fibrosis in HCV infection. comparison with liver biopsy and fibrotest. Hepatology.

[29] Serra-Burriel M., Juanola A., Serra-Burriel F., Thiele M., Graupera I., Pose E., Pera G., Grgurevic I., Caballeria L., Piano S. (2023). Development, validation, and prognostic evaluation of a risk score for long-term liver-related outcomes in the general population: A multicohort study. Lancet.

[30] Karlas T., Petroff D., Sasso M., Fan J.G., Mi Y.Q., de Lédinghen V., Kumar M., Lupsor-Platon M., Han K.H., Cardoso A.C. (2017). Individual patient data meta-analysis of controlled attenuation parameter (CAP) technology for assessing steatosis. J. Hepatol..

[31] Harrison S.A., Taub R., Neff G.W., Lucas K.J., Labriola D., Moussa S.E., Alkhouri N., Bashir M.R. (2023). Resmetirom for nonalcoholic fatty liver disease: A randomized, double-blind, placebo-controlled phase 3 trial. Nat. Med..

[32] Harrison S.A., Bedossa P., Guy C.D., Schattenberg J.M., Loomba R., Taub R., Labriola D., Moussa S.E., Neff G.W., Rinella M.E. (2024). A Phase 3, Randomized, Controlled Trial of Resmetirom in NASH with Liver Fibrosis. N. Engl. J. Med..

[33] Ayada I., van Kleef L.A., Zhang H., Liu K., Li P., Abozaid Y.J., Lavrijsen M., Janssen H.L., van der Laan L.J., Ghanbari M. (2023). Dissecting the multifaceted impact of statin use on fatty liver disease: A multidimensional study. EBioMedicine.

[34] Kuchay M.S., Krishan S., Mishra S.K., Farooqui K.J., Singh M.K., Wasir J.S., Bansal B., Kaur P., Jevalikar G., Gill H.K. (2018). Effect of empagliflozin on liver fat in patients with type 2 diabetes and nonalcoholic fatty liver disease: A randomized controlled trial (E-LIFT trial). Diabetes Care.

[35] Latva-Rasku A., Honka M.-J., Kullberg J., Mononen N., Lehtimäki T., Saltevo J., Kirjavainen A.K., Saunavaara V., Iozzo P., Johansson L. (2019). The SGLT2 inhibitor dapagliflozin reduces liver fat but does not affect tissue insulin sensitivity: A randomized, double-blind, placebo-controlled study with 8-week treatment in type 2 diabetes patients. Diabetes Care.

[36] Ratziu V., de Guevara L., Safadi R., Poordad F., Fuster F., Flores-Figueroa J., Arrese M., Fracanzani A.L., Ben Bashat D., Lackner K. (2021). Aramchol in patients with nonalcoholic steatohepatitis: A randomized, double-blind, placebo-controlled phase 2b trial. Nat. Med..

[37] Friedman S.L., Ratziu V., Harrison S.A. (2018). i wsp.: A randomized, placebo-controlled trial of cenicriviroc for treatment of nonalcoholic steatohepatitis with fibrosis. Hepatology.

[38] Francque S.M., Bedossa P., Ratziu V., Anstee Q.M., Bugianesi E., Sanyal A.J., Loomba R., Harrison S.A., Balabanska R., Mateva L. (2021). A Randomized, Controlled Trial of the Pan-PPAR Agonist Lanifibranor in NASH. N. Engl. J. Med..

[39] Gawrieh S., Noureddin M., Loo N., Mohseni R., Awasty V., Cusi K., Kowdley K.V., Lai M., Schiff E., Parmar D. (2021). Saroglitazar, a PPAR-α/γ Agonist, for Treatment of NAFLD: A Randomized Controlled Double-Blind Phase 2 Trial. Hepatology.

[40] Loomba R., Kayali Z., Noureddin M., Ruane P., Lawitz E.J., Bennett M., Wang L., Harting E., Tarrant J.M., McColgan B.J. (2018). GS-0976 Reduces Hepatic Steatosis and Fibrosis Markers in Patients with Nonalcoholic Fatty Liver Disease. Gastroenterology.

[41] Harrison S.A., Wong V.W., Okanoue T., Bzowej N., Vuppalanchi R., Younes Z., Kohli A., Sarin S., Caldwell S.H., Alkhouri N. (2020). Selonsertib for patients with bridging fibrosis or compensated cirrhosis due to NASH: Results from randomized phase III STELLAR trials. J. Hepatol..

[42] Cheng C., Tsuneyama K., Kominami R., Shinohara H., Sakurai S., Yonekura H., Watanabe T., Takano Y., Yamamoto H., Yamamoto Y. (2005). Expression profiling of endogenous secretory receptor for advanced glycation end products in human organs. Mod. Pathol..

[43] Dimova R., Chakarova N., Grozeva G., Tankova T. (2021). The relationship between endogenous secretory RAGE and cardiac autonomic function in prediabetes. Int. J. Clin. Pract..

[44] Shimizu Y., Harashima A., Munesue S., Oishi M., Hattori T., Hori O., Kitao Y., Yamamoto H., Leerach N., Nakada M. (2020). Neuroprotective Effects of Endogenous Secretory Receptor for Advanced Glycation End-products in Brain Ischemia. Aging Dis..

[45] Semba R.D., Cotch M.F., Gudnason V., Eiríksdottir G., Harris T.B., Sun K., Klein R., Jonasson F., Ferrucci L., Schaumberg D.A. (2014). Serum carboxymethyllysine, an advanced glycation end product, and age-related macular degeneration: The Age, Gene/Environment Susceptibility-Reykjavik Study. JAMA Ophthalmol..

[46] Twarda-Clapa A., Olczak A., Białkowska A.M., Koziołkiewicz M. (2022). Advanced Glycation End-Products (AGEs): Formation, Chemistry, Classification, Receptors, and Diseases Related to AGEs. Cells.

[47] Bijnen M., van Greevenbroek M.M.J., van der Kallen C.J.H., Scheijen J.L., van de Waarenburg M.P.H., Stehouwer C.D.A., Wouters K., Schalkwijk C.G. (2019). Hepatic Fat Content and Liver Enzymes Are Associated with Circulating Free and Protein-Bound Advanced Glycation End Products, Which Are Associated with Low-Grade Inflammation: The CODAM Study. J. Diabetes Res..

[48] Litwinowicz K., Waszczuk E., Gamian A. (2021). Advanced Glycation End-Products in Common Non-Infectious Liver Diseases: Systematic Review and Meta-Analysis. Nutrients.

[49] Nagata C., Wada K., Yamakawa M., Nakashima Y., Koda S., Uji T., Oba S. (2020). Dietary Intake of Nε-carboxymethyl-lysine, a Major Advanced Glycation End Product, is Not Associated with Increased Risk of Mortality in Japanese Adults in the Takayama Study. J. Nutr..

[50] Sharma C., Kaur A., Thind S.S., Singh B., Raina S. (2015). Advanced glycation End-products (AGEs): An emerging concern for processed food industries. J. Food Sci. Technol..

[51] Rungratanawanich W., Qu Y., Wang X., Essa M.M., Song B.J. (2021). Advanced glycation end products (AGEs) and other adducts in aging-related diseases and alcohol-mediated tissue injury. Exp. Mol. Med..

[52] Zhao J., Randive R., Stewart J.A. (2014). Molecular mechanisms of AGE/RAGE-mediated fibrosis in the diabetic heart. World J. Diabetes.

[53] Priken K., Tapia G., Cadagan C., Quezada N., Torres J., D’Espessailles A., Pettinelli P. (2022). Higher hepatic advanced glycation end products and liver damage markers are associated with nonalcoholic steatohepatitis. Nutr. Res..

[54] Rahbar S., Blumenfeld O., Ranney H.M. (1969). Studies of an Unusual Hemoglobin in Patients with Diabetes Mellitus. Biochem. Biophys. Res. Commun..

[55] Nentwich M.M. (2015). Diabetic Retinopathy—Ocular Complications of Diabetes Mellitus. World J. Diabetes.

[56] Yang Z., Lou X., Zhang J., Nie R., Liu J., Tu P., Duan P. (2021). Association Between Early Markers of Renal Injury and Type 2 Diabetic Peripheral Neuropathy. Diabetes. Metab. Syndr. Obes..

[57] Sharifi-Zahabi E., Sharafabad F.H., Abdollahzad H., Malekahmadi M., Rad N.B. (2021). Circulating Advanced Glycation End Products and Their Soluble Receptors in Relation to All-Cause and Cardiovascular Mortality: A Systematic Review and Meta-analysis of Prospective Observational Studies. Adv. Nutr..

[58] Asadipooya K., Lankarani K.B., Raj R., Kalantarhormozi M. (2019). RAGE is a Potential Cause of Onset and Progression of Nonalcoholic Fatty Liver Disease. Int. J. Endocrinol..

[59] Palma-Duran S.A., Kontogianni M.D., Vlassopoulos A., Zhao S., Margariti A., Georgoulis M., Papatheodoridis G., Combet E. (2018). Serum levels of advanced glycation end-products (AGEs) and the decoy soluble receptor for AGEs (sRAGE) can identify non-alcoholic fatty liver disease in age-, sex- and BMI-matched normo-glycemic adults. Metabolism.

[60] Mehta R., Shaw G., Masschelin P., Felix S., Otgonsuren M., Baranova A., Goodman Z., Younossi Z. (2018). Polymorphisms in the receptor for advanced glycation end-products (RAGE) gene and circulating RAGE levels as a susceptibility factor for non-alcoholic steatohepatitis (NASH). PLoS ONE.

[61] Cao X., Xiao X., Jiang P., Fu N. (2025). Construction and evaluation of a diagnostic model for metabolic dysfunction-associated steatotic liver disease based on advanced glycation end products and their receptors. Front. Med..

[62] Ivancovsky-Wajcman D., Zelber-Sagi S., Fliss Isakov N., Webb M., Zemel M., Shibolet O., Kariv R. (2019). Serum Soluble Receptor for AGE (sRAGE) Levels Are Associated With Unhealthy Lifestyle and Nonalcoholic Fatty Liver Disease. Clin. Transl. Gastroenterol..

[63] Laudenslager M., Lazo M., Wang D., Selvin E., Chen P.H., Pankow J.S., Clark J.M. (2021). Association between the soluble receptor for advanced glycation end products (sRAGE) and NAFLD in participants in the Atherosclerosis Risk in Communities Study. Dig. Liver Dis..

[64] Hellwig M., Auerbach C., Müller N., Samuel P., Kammann S., Beer F., Gunzer F., Henle T. (2019). Metabolization of the Advanced Glycation End Product N-ε-Carboxymethyllysine (CML) by Different Probiotic *E. coli* Strains. J. Agric. Food Chem..

[65] Musso G., Saba F., Cassader M., Gambino R. (2023). Lipidomics in pathogenesis, progression and treatment of nonalcoholic steatohepatitis (NASH): Recent advances. Prog. Lipid Res..

[66] Masoodi M., Gastaldelli A., Hyötyläinen T., Arretxe E., Alonso C., Gaggini M., Brosnan J., Anstee Q.M., Millet O., Ortiz P. (2021). Metabolomics and lipidomics in NAFLD: Biomarkers and non-invasive diagnostic tests. Nat. Rev. Gastroenterol. Hepatol..

[67] Feldstein A.E., Lopez R., Tamimi T.A., Yerian L., Chung Y.M., Berk M., Zhang R., McIntyre T.M., Hazen S.L. (2010). Mass spectrometric profiling of oxidized lipid products in human nonalcoholic fatty liver disease and nonalcoholic steatohepatitis. J. Lipid Res..

[68] Caussy C., Chuang J.C., Billin A., Hu T., Wang Y., Subramanian G.M., Djedjos C.S., Myers R.P., Dennis E.A., Loomba R. (2020). Plasma eicosanoids as noninvasive biomarkers of liver fibrosis in patients with nonalcoholic steatohepatitis. Ther. Adv. Gastroenterol..

[69] Puri P., Wiest M.M., Cheung O., Mirshahi F., Sargeant C., Min H., Contos M.J., Sterling R.K., Fuchs M., Zhou H. (2009). The plasma lipidomic signature of nonalcoholic steatohepatitis. Hepatology.

[70] Quehenberger O., Armando A.M., Cedeno T.H., Loomba R., Sanyal A.J., Dennis E.A. (2024). Novel eicosanoid signature in plasma provides diagnostic for metabolic dysfunction-associated steatotic liver disease. J. Lipid Res..

[71] Meikle T.G., Huynh K., Giles C., Meikle P.J. (2021). Clinical lipidomics: Realizing the potential of lipid profiling. J. Lipid Res..

[72] Elhoseeny M.M., Abdulaziz B.A., Mohamed M.A., Elsharaby R.M., Rashad G.M., Othman A.A.A. (2024). Fetuin-A: A relevant novel serum biomarker for non-invasive diagnosis of metabolic dysfunction-associated steatotic liver disease (MASLD): A retrospective case-control study. BMC Gastroenterol..

[73] Haukeland J.W., Dahl T.B., Yndestad A., Gladhaug I.P., Løberg E.M., Haaland T., Konopski Z., Wium C., Aasheim E.T., Johansen O.E. (2012). Fetuin A in nonalcoholic fatty liver disease: In vivo and in vitro studies. Eur. J. Endocrinol..

[74] Pan X., Kaminga A.C., Chen J., Luo M., Luo J. (2020). Fetuin-A and Fetuin-B in Non-Alcoholic Fatty Liver Disease: A Meta-Analysis and Meta-Regression. Int. J. Environ. Res. Public Health.

[75] Zhang P., Qi Z., Xu H., Zhou L., Zhao X., Zhong H., Zhou W., Fan B., Wu H., Ge J. (2025). Fetuin-A increases thrombosis risk in non-alcoholic fatty liver disease by binding to TLR-4 on platelets. Cardiovasc. Res..

[76] Liu S., Xiao J., Zhao Z., Wang M., Wang Y., Xin Y. (2021). Systematic Review and Meta-analysis of Circulating Fetuin-A Levels in Nonalcoholic Fatty Liver Disease. J. Clin. Transl. Hepatol..

[77] Sato M., Kamada Y., Takeda Y., Kida S., Ohara Y., Fujii H., Akita M., Mizutani K., Yoshida Y., Yamada M. (2015). Fetuin-A negatively correlates with liver and vascular fibrosis in nonalcoholic fatty liver disease subjects. Liver Int..

[78] Frei A., Zimmermann A., Weigand K. (1984). The N-terminal propeptide of collagen type III in serum reflects activity and degree of fibrosis in patients with chronic liver disease. Hepatology.

[79] Celebi G., Genc H., Gurel H., Sertoglu E., Kara M., Tapan S., Acikel C., Karslioglu Y., Ercin C.N., Dogru T. (2015). The relationship of circulating fetuin-a with liver histology and biomarkers of systemic inflammation in nondiabetic subjects with nonalcoholic fatty liver disease. Saudi J. Gastroenterol..

[80] Kamada Y., Miyoshi E. (2015). Value of fetuin-A as a predictor of liver fibrosis in patients with nonalcoholic fatty liver disease. Author’s reply. Liver Int..

[81] Teare J.P., Sherman D., Greenfield S.M., Simpson J., Bray G., Catterall A.P., Murray-Lyon I.M., Peters T.J., Williams R., Thompson R.P. (1993). Comparison of serum procollagen III peptide concentrations and PGA index for assessment of hepatic fibrosis. Lancet.

[82] Rohde H., Vargas L., Hahn E., Kalbfleisch H., Bruguera M., Timpl R. (1979). Radioimmunoassay for type III procollagen peptide and its application to human liver disease. Eur. J. Clin. Invest..

[83] Zachariae H., Heickendorff L., Sogaard H. (2001). The value of amino-terminal propeptide of type III procollagen in routine screening for methotrexate-induced liver fibrosis: A 10-year follow-up. Br. J. Dermatol..

[84] Nielsen M.J., Nedergaard A.F., Sun S., Veidal S.S., Larsen L., Zheng Q., Suetta C., Henriksen K., Christiansen C., Karsdal M.A. (2013). The neo-epitope specific PRO-C3 ELISA measures true formation of type III collagen associated with liver and muscle parameters. Am. J. Transl. Res..

[85] Caussy C., Bhargava M., Villesen I.F., Gudmann N.S., Leeming D.J., Karsdal M.A., Faulkner C., Bao D., Liu A., Lo M.T. (2019). Collagen Formation Assessed by N-Terminal Propeptide of Type 3 Procollagen Is a Heritable Trait and Is Associated with Liver Fibrosis Assessed by Magnetic Resonance Elastography. Hepatology.

[86] Mak A.L., Lee J., van Dijk A.M., Vali Y., Aithal G.P., Schattenberg J.M., Anstee Q.M., Brosnan M.J., Zafarmand M.H., Ramsoekh D. (2021). Systematic review with meta-analysis: Diagnostic accuracy of Pro-C3 for hepatic fibrosis in patients with non-alcoholic fatty liver disease. Biomedicine.

[87] Luo Y.I., Oseini A., Gagnon R., Charles E.D., Sidik K., Vincent R., Collen R., Idowu M., Contos M.J., Mirshahi F. (2018). An Evaluation of the Collagen Fragments Related to Fibrogenesis and Fibrolysis in Nonalcoholic Steatohepatitis. Sci. Rep..

[88] Liu M., Qiu H., Zhang W., Mei T., Tang S., Gao Y., Zhu Y., Huang X., Yu H. (2023). Evaluation of blood-based PRO-C3 testing as a diagnostic marker for staging liver fibrosis: A systematic review and meta-analysis. J. Gastroenterol. Hepatol..

[89] Daniels S.J., Leeming D.J., Eslam M., Hashem A.M., Nielsen M.J., Krag A., Karsdal M.A., Grove J.I., Neil Guha I., Kawaguchi T. (2019). ADAPT: An Algorithm Incorporating PRO-C3 Accurately Identifies Patients with NAFLD and Advanced Fibrosis. Hepatology.

[90] Nielsen M.J., Leeming D.J., Goodman Z., Friedman S., Frederiksen P., Rasmussen D.G.K., Vig P., Seyedkazemi S., Fischer L., Torstenson R. (2021). Comparison of ADAPT, FIB-4 and APRI as non-invasive predictors of liver fibrosis and NASH within the CENTAUR screening population. J. Hepatol..

[91] Tang L.J., Li G., Eslam M., Zhu P.W., Chen S.D., Leung H.H., Huang O.Y., Wong G.L., Zhou Y.J., Karsdal M. (2023). N-terminal propeptide of type 3 collagen-based sequential algorithm can identify high-risk steatohepatitis and fibrosis in MAFLD. Hepatol. Int..

[92] Madsen B.S., Thiele M., Detlefsen S., Kjaergaard M., Møller L.S., Trebicka J., Nielsen M.J., Gudmann N.S., Leeming D.J., Karsdal M.A. (2021). PRO-C3 and ADAPT algorithm accurately identify patients with advanced fibrosis due to alcohol-related liver disease. Aliment. Pharmacol. Ther..

[93] Babu M., Snyder M. (2023). Multi-Omics Profiling for Health. Mol. Cell Proteom..

[94] Nurk S., Koren S., Rhie A., Rautiainen M., Bzikadze A.V., Mikheenko A., Vollger M.R., Altemose N., Uralsky L., Gershman A. (2022). The complete sequence of a human genome. Science.

[95] Jiang L., Wang M., Lin S., Jian R., Li X., Chan J., Dong G., Fang H., Robinson A.E., GTEx Consortium (2020). A Quantitative Proteome Map of the Human Body. Cell.

[96] Luo Y., Wadhawan S., Greenfield A., Decato B.E., Oseini A.M., Collen R., Shevell D.E., Thompson J., Jarai G., Charles E.D. (2021). SOMAscan Proteomics Identifies Serum Biomarkers Associated with Liver Fibrosis in Patients With NASH. Hepatol. Commun..

[97] Sveinbjornsson G., Ulfarsson M.O., Thorolfsdottir R.B., Jonsson B.A., Einarsson E., Gunnlaugsson G., Rognvaldsson S., Arnar D.O., Baldvinsson M., Bjarnason R.G. (2022). Multiomics study of nonalcoholic fatty liver disease. Nat. Genet..

[98] Langfelder P., Horvath S. (2008). WGCNA: An R package for weighted correlation network analysis. BMC Bioinform..

[99] Zhang J.J., Shen Y., Chen X.Y., Jiang M.L., Yuan F.H., Xie S.L., Zhang J., Xu F. (2023). Integrative network-based analysis on multiple Gene Expression Omnibus datasets identifies novel immune molecular markers implicated in non-alcoholic steatohepatitis. Front. Endocrinol..

[100] Zhang B., Horvath S. (2005). A general framework for weighted gene co-expression network analysis. Stat. Appl. Genet. Mol. Biol..

[101] Wishart D.S., Guo A., Oler E., Wang F., Anjum A., Peters H., Dizon R., Sayeeda Z., Tian S., Lee B.L. (2022). HMDB 5.0: The human metabolome database for 2022. Nucleic Acids Res..

[102] Evans W., Meslin E.M., Kai J., Qureshi N. (2024). Precision Medicine-Are We There Yet? A Narrative Review of Precision Medicine’s Applicability in Primary Care. J. Pers. Med..

[103] Denny J.C., Collins F.S. (2021). Precision medicine in 2030-seven ways to transform healthcare. Cell.

